# Analysis of matched geographical areas to study potential links between environmental exposure to oil refineries and non-Hodgkin lymphoma mortality in Spain

**DOI:** 10.1186/1476-072X-11-4

**Published:** 2012-02-06

**Authors:** Rebeca Ramis, Peter Diggle, Elena Boldo, Javier Garcia-Perez, Pablo Fernandez-Navarro, Gonzalo Lopez-Abente

**Affiliations:** 1 Department of Environmental Epidemiology and Cancer, National Centre for Epidemiology, Carlos III Institute of Health, Madrid, Spain; 2 Consortium for Biomedical Research in Epidemiology & Public Health (CIBER en Epidemiología y Salud Pública--CIBERESP), Madrid, Spain; 3 Faculty of Health and Medicine, Lancaster University, Lancaster, UK

**Keywords:** Non-Hodgkin lymphoma, Refinery, Pollution, Mortality, Matched analysis

## Abstract

**Background:**

Emissions from refineries include a wide range of substances, such as chrome, lead, nickel, zinc, arsenic, cadmium, benzene, dioxins and furans, all of which are recognized by the International Agency for Research on Cancer (IARC) as carcinogens.

Various studies have shown an association between non-Hodgkin lymphoma (NHL) and residence in the vicinity of industrial areas; however, evidence of specific association between refineries and residence in the vicinity has been suggested but not yet established.

The aim of this study is to investigate potential links between environmental exposure to emissions from refineries and non-Hodgkin lymphoma mortality in Spain.

The spatial distribution of NHL in Spain has an unusual pattern with regions some showing higher risk than others.

**Methods:**

We designed an analysis of matched geographical areas to examine non-Hodgkin lymphoma mortality in the vicinity of the 10 refineries sited in Spain over the period 1997-2006. Population exposure to refineries was estimated on the basis of distance from town of residence to the facility in a 10 km buffer.

We defined 10 km radius areas to perform the matching, accounting for population density, level of industrialization and socio-demographic factors of the area using principal components analysis.

For the matched towns we evaluated the risk of NHL mortality associated with residence in the vicinity of the refineries and with different regions using mixed Poisson models. Then we study the residuals to assess a possible risk trend with distance.

**Results:**

Relative risks (RRs) associated with exposure showed similar values for women and for men, 1.09 (0.97-1.24) and 1.12 (0.99-1.27). RRs for two regions were statistically significant: Canary Islands showed an excess of risk of 1.35 (1.05-1.72) for women and 1.50 (1.18-1.92) for men, whilst Galicia showed an excess of risk of 1.35 (1.04-1.75) for men, but not significant excess for women.

**Conclusions:**

The results suggest a possible increased risk of NHL mortality among populations residing in the vicinity of refineries; however, a potential distance trend has not been shown. Regional effects in the Canary Islands and Galicia are significantly greater than the regional average.

## Background

Emissions from refineries include a wide range of substances such as chrome, lead, nickel, zinc, arsenic, cadmium, benzene, dioxins and furans, all of which are recognized by the International Agency for Research on Cancer (IARC) [1] as carcinogens. Concern about these emissions and their effect on cancer has been studied in several countries such as Sweden, the United States, Italy, the United Kingdom and Taiwan. In many cases the results of those studies suggested association but without statistical significance. A resent Swedish study over a region with high concentration of petrochemical industries showed no excess of risk for lung, leukemia, lymphoma, liver or central nervous system cancer [2]. The study used small subareas that were classified as "low" or "high" exposed areas according to monitored measures of pollutants. Another recent study in the United States conducted a matched case-control analysis to study lung cancer in a region of Louisiana with high concentration of petrochemical industries [3]. Exposure was approximated by 3 computed buffers at 0.5 miles, 1 mile and 2 miles. Although cases were more likely to have lived close to a petrochemical site no significant association was established. In Italy another case-control study in the vicinity of a petrochemical plant located in Brindisi showed moderate increases in risk for lung, bladder and lymphohematopoietic neoplasms among the population resident within 2 km from the site [4]; however, those increases were not statistically significant. In the UK, Wilkinson et al. [5] conducted a study to analyse the incidence of lymphohematopoietic malignancy at small area level within 7.5 km from 11 oil refineries. No evidence of association between residence in the vicinity of the oil refineries and increase in incidence was found. In another study in the UK, a region with large industrial activity including petrochemical complexes was compared with a region with no industry but similar socio-economical characteristics [6]. The industrialized area showed an increase in risk of lung cancer in women. And in Taiwan, Yang et al. [7] reported increased incidence of liver cancer.

The association between non-Hodgkin lymphoma (NHL) and residence in the vicinity of industrial areas has been analyzed in various studies. A case-control study conducted in Canada showed for women increased risk for NHL with proximity to copper smelters and sulphite pulp mills [8]. A study of NHL mortality in Spain showed increased risk associated with proximity to paper and pulp industry [9]. A recent study in France found increased NHL risk among people living near to solid waste incinerators [10]. Several studies conducted in the US specifically evaluated petrochemical industries. One case-control study [11] evaluated the risk of NHL associated with residence within 2 miles of industrial facilities. Residence near refineries showed an association with risk for follicular lymphoma though it was not statistically significant. A previous case-control study [12] found a statistically significant increase in risk of NHL for those living near industrial facilities; however, specific association with petrochemical industry was not statistically significant.

In Spain the concern about pollutant emissions from petrochemical plants has motivated several studies focused in regions with high concentration of chemical and petrochemical sites in the east and south of the Iberian Peninsula [13-15]. However, to the best of our knowledge this is the first study that analyses all the Spanish refineries jointly in the same model in relation to health outcomes.

The aim of this study is to investigate potential links between environmental exposure to emissions from refineries and non-Hodgkin lymphoma mortality in Spain; for that purpose we designed and conducted an ecological study using matched geographic areas to control for potential confounders.

## Methods

### Studied period, mortality and population data

Observed municipal mortality data corresponding to deaths coded as non-Hodgkin lymphoma were drawn from the records of the National Statistics Institute (*INE*) over the period 1997-2006 for the 8098 Spanish municipalities; codes 200, 202 under International Classification of Diseases-9^th ^Revision (ICD-9) and C82-C84, C96 (ICD-10). Expected cases were calculated by taking the specific rates for Spain as a whole, broken down by age group (18 groups, 0-4, 5-9,...,85 and over), sex, and five-year period (1997-2001, 2002-2006), and multiplying these by the person-years for each town, broken down by the same strata. For calculation of person-years, the two five-year periods were considered separately, with data corresponding to 1999 and 2004 taken as the estimator of the population for each five-year period.

**Figure 1:**
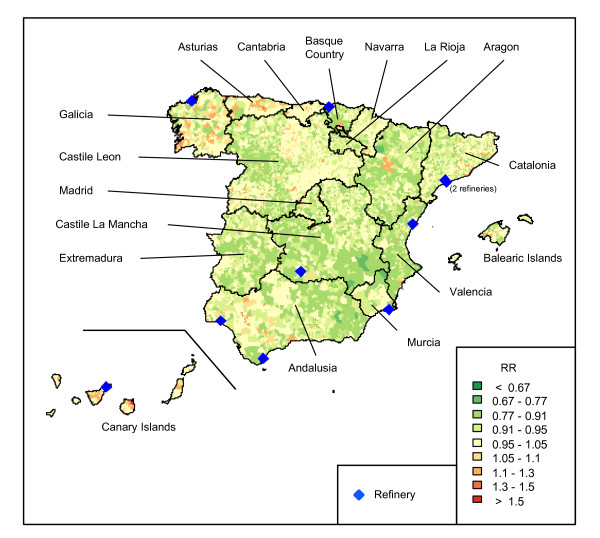
**Map of RR at municipal level. Regions boundaries black lines**. Refinery locations in blue.

The spatial distribution of NHL mortality risk presented in a previous cancer atlas showed a characteristic pattern with high risk and low risk regions [16]. To include this regional variability in the analysis we used a regional covariate named *CCAA *(Figure 1).

### Industrial facilities information

Information about the industrial facilities was obtained from the European Pollutant Release and Transfer Register (E-PRTR) created by the European Commission (E-PRTR 2007). This register makes it compulsory for European industrial facilities to declare all emissions that exceed the designated thresholds. It gathers information on releases, industrial activities and waste management. E-PRTR records thus constitute a public inventory of industries that is a valuable resource for monitoring industrial pollution [9,17,18]. According to the Spanish register, PRTR-Spain (Ministry for the Environment and Rural & Marine Habitats, 2007), there were 10 refineries operating in Spain in 2007. Figure 1 shows a map with the location of the 10 refineries. The first started to operate in 1930 and the last in 1974; however most of them started to operate in the 1960s.

During the year 2007 the Spanish refineries released around 100,000 tonnes of pollutants, half of which are classified as hazardous, and many released substances associated with cancer by the IARC, such as chrome, lead, nickel, zinc, arsenic, cadmium, benzene, dioxins and furans. A summary of released substances is given in the Additional file 1.

### Socio-demographic covariates

Socio-demographic data was obtained from the 1991 census. Particular covariates were chosen for their availability at municipal level and potential explanatory ability over certain geographic mortality patterns. The chosen covariates were: population density; percentage of illiteracy (*illiteracy*), percentage of unemployed (*unemployed*); percentage of farmers (*farmers*); percentage of over 65 (*p65*); average persons per household according to the 1991 census (*pph*); and mean income as a measure of income level (*income*) [19]. We used the 1991 census, previous to the studied period to account for the latency period [20]. Before their inclusion in the model the covariates were standardized.

### Exposure coding

We constructed metrics for residential proximity to the 10 refineries. We computed the distance between each municipality and each industrial site considering the centroid as measurement point in both cases. We considered the municipal centroid to be the centre of the town and refinery centroid to be the central point of the facility. We defined as exposed those municipalities with maximum distance from a site of 10 km and not exposed the remaining municipalities (variable *Expo*).

### Exploratory analysis

As a first approach we performed an exploratory analysis over the 8098 municipalities. We fitted a Besag, York and Molliè (BYM) model. This model is based in a Poisson regression with an unstructured random effect and a spatial random effect to account for the spatial structures of the data [21]. We included the exposure variable (*Expo*) as well as the socio-demographic covariates (*Soc*) and region (CCAA). Relative risks (RRs) and their 95% credible intervals (95% CIs) were estimated for the covariates.

Model 1

$$O_{i}\sim Poisson\;\left(\mu_{i}=E_{i}\lambda_{i}\right)$$

$$\begin{array}{c} \text{log}\;\text{(}\lambda_{i}\text{)}=\rho +\alpha_{i}*Expo_{i}+\underset{j}{\sum }\beta_{j}Soc_{ij}+CCAA_{i}+h_{i}+b_{i}\\ \Rightarrow \text{log}\;\left(\mu_{i}\right)=\text{log}\;\text{(}E_{i}\text{)}+\rho +\alpha_{i}*Expo_{i}+\underset{j}{\sum }\beta_{j}Soc_{ij}+CCAA_{i}+h_{i}+b_{i} \end{array}$$

$$\begin{array}{c} h_{i}\sim Normal\;\left(0,\tau_{h}\right)\\ b_{i}\sim Car\text{.}Normal\;\left(\eta_{i},\tau_{b}\right) \end{array}$$

Where *i *is the municipality and *j *the covariate

### Area matching

In a second stage we performed a matched analysis; for this purpose we first defined geographical areas as follows. The exposed areas (*Expo*) were defined around the 10 refineries using a 10 km buffer; the centre of the buffer was the centre of the refinery. Each of these 10 exposed areas included all the municipalities whose centroids were located within the 10 km buffer. We aggregated the mortality figures and computed new values for socio-demographic covariates. We then constructed a 10 km radius buffer around each Spanish municipality to define the non-exposed areas, giving 8098 overlapping areas. For each of the areas we aggregated the mortality figures from the contained municipalities and computed new values for socio-demographic covariates using data from the municipal socio-demographic covariates and combining them as the means weighted by population sizes.

**Figure 2:**
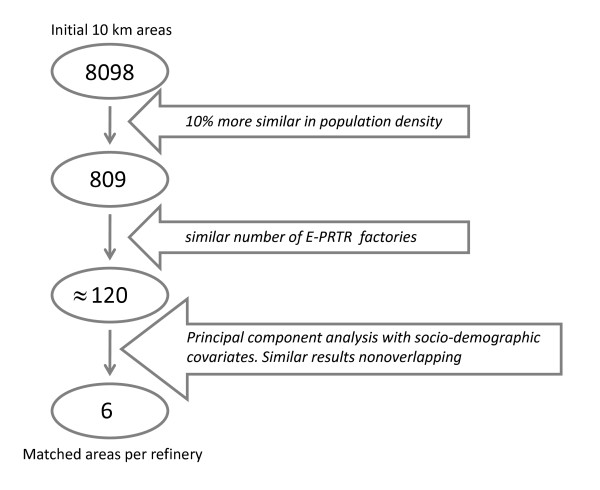
**Diagram of the matching strategy**.

For each of the exposed areas we then selected a small number of non-exposed areas by matching. We carried out the matching according to similarity of socio-demographic and industrial characteristics. We used the following strategy to select the matched areas for each exposed area (Figure 2). Initially, we selected the 10% (809) most similar non-exposed areas according to the population density. Then, among these we selected around 15% of the areas with equal or very similar number of PRTR industries. Next, over the remaining areas we performed a principal component analysis using the socio-demographic covariates; based on these results we selected the 6 most similar areas (or 8 areas for the exposed area with the highest population density) with the restriction of their not overlapping so as to avoid having the same municipality in two different areas. Finally, we defined 10 matching groups, one for each exposed area (refinery); each of these matching groups had one exposed area and 6 (or 8) non-exposed areas. We then defined a new variable accounting for the matching group (*Group*).

### Matched areas analysis

For this analysis we fitted a Poisson regression with mixed effects (Model 2) to the data from the 72 matched areas. The variables included in the model were the exposure (Expo), socio-economic covariates (Soc), region (CCAA) as fixed effects and matching group (Group) variables as random effect. RRs and their 95% CIs were estimated.

Model 2

$$O_{gi}\sim Poisson\;\left(\mu_{gi}=E_{gi}\lambda_{gi}\right)$$

$$\text{log}\;\text{(}\lambda_{gi}\text{)}=\rho +\alpha_{gi}*Expo_{i}+\underset{j}{\sum }\beta_{j}Soc_{gij}+CCAA_{gi}+Group_{gi}$$

$$Group_{i}\sim Normal\;\left(0,\tau_{h}\right)$$

Where *g *is the matching group, *i *is the municipality and *j *the covariate

Finally, we studied possible changes (gradient) in the mortality risk distribution with increasing distance between the refineries and the municipal centroids. We fitted Model 2 again but without the exposure variable (*Expo*). Then we assessed the behaviour of the residuals with increasing distance and compared results for the exposed and non-exposed municipalities. To be able to do the comparison we needed distances for the non-exposed areas; therefore, we constructed artificial distances. We assumed that each of the 62 non-exposed areas had a refinery in its centre and we computed the distance from each municipal centroid to this imaginary point source. Then we used these distances and the distances to the real refineries in the exposed areas to fit non-parametric smoothers to the residuals (lowess) [22].

We fitted all models for men and women separately. We fitted the BYM and mixed models by Integrated Nested Laplace Approximations (INLAs) [23], using the R package R-INLA. We fitted the non-parametric smoothers by local polynomial regression function *loess *from the R package *stats*.

## Results

For the studied period a total of 12,229 men and 11,109 women died of non-Hodgkin lymphoma in Spain, out of population in excess of 40 million. Table 1 presents specific data regarding the exposed areas and the refineries. As already mentioned, all 10 refineries have been working for decades. The total number of municipalities within the 10 km buffers around the 10 refineries was 77, varying from 2 to 24 per refinery; the mean population ranged from 28,357 to 354,351 with a total of 1,744,988; and the number of E-PRTR factories in the vicinity of each of the municipal centroids varied from 0 to 55. According to the data, a total around 1% (77/8098) of the municipalities were classified as exposed, corresponding to around 4.3% of the population. Regarding the number of cases, 675 (5.52%) men and 614 (5.53%) women fell in the exposed category. The standardized mortality ratios (SMR = obs/exp) were greater than 1 in the majority of the areas, with overall values of 1.24 for men and 1.22 for women.

**Table 1:** Refineries

E-PRTR Code	Municipality	Region	year	**N. Muni**.	**Pop**.	Pop. density	N. Fact	Men				Women				Gr
									
								Obs	Esp	SMR	CI	Obs	Esp	SMR	CI	
1479	San Roque	Andalusia	1967	2	81591	447.8	3	15	20.4	0.74	(0.42-1.18)	17	18.5	0.92	*(0.55-1.44)*	1
704	Castellón de la Plana	Valencia	1967	5	189660	1059.8	27	47	58.4	0.80	(0.59-1.06)	41	51.9	0.79	*(0.57-1.06)*	2
1528	Puertollano	Castilla - La Mancha	1966	4	63525	36.8	0	24	20.1	1.20	(0.78-1.75)	18	18.1	0.99	*(0.60-1.54)*	3
1482	Palos de la Frontera	Andalusia	1967	4	177622	130.5	2	56	40.6	1.38	(1.05-1.77)	51	37.7	1.35	*(1.01-1.76)*	4
1529	Cartagena	Murcia	1951	2	202523	25.6	2	52	51.2	1.01	(0.76-1.32)	48	45.7	1.05	*(0.78-1.38)*	5
1480	Santa Cruz de Tenerife	Canary Islands	1930	2	354351	849.8	2	126	83.5	1.51	(1.26-1.79)	125	75.8	1.65	*(1.37-1.95)*	6
1527	Pobla de Mafumet	Catalonia	1974	19	28357	3687.7	33	80	67.0	1.19	(0.95-1.48)	60	59.5	1.01	*(0.77-1.28)*	7
1577	Tarragona	Catalonia	1968	24	98625	1444.7	34	50	40.3	1.24	(0.93-1.62)	42	35.3	1.19	*(0.86-1.59)*	8
3701	Muskiz	Basque Country	1970	11	255936	4264.7	55	80	74.4	1.07	(0.86-1.33)	81	69.9	1.16	*(0.92-1.43)*	9
1526	La Coruña	Galicia	1964	4	292798	757.8	4	145	89.6	1.62	(1.37-1.90)	131	89.2	1.47	*(1.23-1.73)*	10

*Total*	*-*	*-*	*-*	*77*	*1744988*	*-*	*-*	*675*	*545*	*1.24*	*(1.14-1.33)*	*614*	*501.5*	*1.22*	*(1.13-1.32)*	*-*

First four columns contain information about each refinery such as E-PRTR code, municipality and region, and the starting year. Next four columns to the right give information on the areas: number of municipalities included (N. Muni); population based on 1999 census (Population); population density based on 1999 census, habitants/km2 (Population density); Number of PRTR factories in the vicinity of each of the municipal centroids (N. fact). Next six columns give the number of observed cases, expected cases and the standardized mortality ratio (SMR) for both men and women. Last column contains the regional variable "group"

For both models the reference region for the CCAA variable was Andalusia. We made this choice for two reasons: firstly, the SMR for Andalusia was the closest to one (SMR = 0.97); secondly, Andalusia is the biggest Spanish region. Table 2 shows the estimated RR and their 95% CIs for both analyses. The two left hand side columns show the results for the spatial analysis with BYM model (Model 1) whilst the two right hand side columns show the results for the matched analysis. For the spatial model, RRs for the exposure variable (*Expo*) showed very similar values for women and for men, 1.13 (1.01-1.26) and 1.12 (1.00-1.26). RRs for three regions were statistically significant higher than one, Canary Islands showed an excess of risk of 1.44 (1.27-1.63) for women and 1.41 (1.25-1.59) for men, Galicia showed an excess of risk of 1.25 (1.12-1.39) for women and 1.39 (1.25-1.54) for men, and Asturias showed an excess of risk of 1.35 (1.16-1.58) for men. There were also regions with RRs statistically significant lower than one, such as Madrid with 0.8 (0.7-0.92) for women and 0.79 (0.69-0.91) for men, and Extremadura with 0.83 (0.72-0.96) for men.

**Table 2:** Relative risks (RR) and 95% credible intervals (95% CIs) for men and women for the spatial analysis and matched analysis.

	Spatial analysis. Model 18098 municipalities				Matched analysis. Model 2 528 municipalities			
		
Fixed effects	Women		Men		Women		Men	
				
	RR	95%CI	RR	95%CI	RR	95%CI	RR	95%CI
Illiteracy	0.98	(0.97-1.00)	0.99	(0.98-1.01)	0.98	(0.93-1.03)	0.99	(0.95-1.03)
Unemployed	1.00	(1.00-1.01)	1.00	(1.00-1.00)	1.00	(0.99-1.01)	1.00	(0.99-1.01)
Farmers	1.00	(1.00-1.00)	1.00	(1.00-1.00)	1.00	(0.99-1.01)	1.00	(0.99-1.01)
Income	1.01	(0.99-1.04)	1.02	(1.00-1.04)	1.01	(0.94-1.08)	1.04	(0.97-1.11)
P65	0.99	(0.98-1.00)	**0.98**	**(0.98-0.99)**	0.98	(0.96-1.01)	1.01	(0.98-1.03)
pph	1.01	(0.89-1.14)	0.96	(0.86-1.08)	0.78	(0.52-1.15)	0.93	(0.65-1.32)
**Expo**	**1.13**	**(1.01-1.26)**	**1.12**	**(1.00-1.26)**	**1.09**	**(0.97-1.24)**	**1.12**	**(0.99-1.27)**
Aragon	0.92	(0.78-1.09)	0.95	(0.80-1.12)	0.80	(0.20-3.28)	0.29	(0.04-2.06)
**Asturias**	1.10	(0.95-1.29)	**1.35**	**(1.16-1.58)**	0.98	(0.70-1.38)	1.15	(0.82-1.65)
Balearics Isl.	1.03	(0.86-1.24)	1.08	(0.91-1.30)	-		-	
**Canary Islands**	**1.44**	**(1.27-1.63)**	**1.41**	**(1.25-1.59)**	**1.35**	**(1.05-1.72)**	**1.50**	**(1.18-1.92)**
Cantabria	1.01	(0.83-1.23)	1.00	(0.82-1.22)	1.50	(0.80-2.83)	0.86	(0.39-1.91)
Castile La Mancha	0.91	(0.80-1.04)	0.88	(0.78-1.00)	0.87	(0.55-1.38)	0.88	(0.58-1.33)
Castile Leon	0.95	(0.84-1.08)	1.01	(0.89-1.15)	0.75	(0.52-1.09)	1.08	(0.78-1.48)
Catalonia	1.09	(0.98-1.22)	1.09	(0.97-1.21)	0.98	(0.77-1.24)	1.00	(0.79-1.26)
**Valencia**	0.90	(0.80-1.00)	0.89	(0.80-1.00)	0.81	(0.61-1.06)	**0.69**	**(0.53-0.91)**
**Extremadura**	0.98	(0.86-1.13)	**0.83**	**(0.72-0.96)**	0.92	(0.49-1.70)	0.78	(0.42-1.45)
**Galicia**	**1.25**	**(1.12-1.39)**	**1.39**	**(1.25-1.54)**	1.18	(0.91-1.55)	**1.35**	**(1.04-1.75)**
**Madrid**	**0.80**	**(0.70-0.92)**	**0.79**	**(0.69-0.91)**	**0.64**	**(0.42-0.98)**	0.76	(0.51-1.11)
Murcia	0.96	(0.81-1.13)	1.03	(0.88-1.21)	0.88	(0.61-1.28)	0.92	(0.64-1.33)
Navarra	0.86	(0.70-1.07)	0.83	(0.67-1.02)	0.72	(0.51-1.03)	1.08	(0.79-1.48)
Basque Country	0.99	(0.87-1.12)	1.07	(0.94-1.22)	0.92	(0.73-1.16)	0.87	(0.69-1.10)
Rioja	0.82	(0.61-1.09)	0.87	(0.66-1.14)	0.64	(0.20-2.05)	0.97	(0.39-2.42)

p65: percentage of over 65; pph: average persons per household

**Figure 3:**
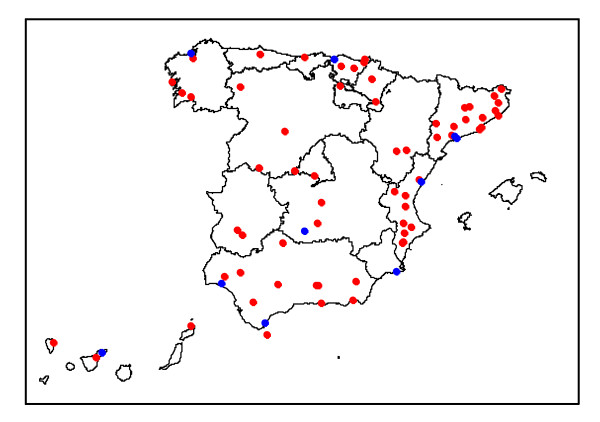
**Map of the areas**. Refineries in blue dots and the non-exposed areas in red dots.

The matching strategy produced a sample of 72 areas, 62 non-exposed areas to add to the 10 exposed areas, giving a total of 528 municipalities for the matched analysis (77 exposed and 451 non-exposed). Figure 3 presents a map with the areas' locations; the exposed areas (refineries) are shown as blue dots and the matched non-exposed areas as red dots. The two right hand side columns of Table 2 show the results for the matched analysis. Resulting RRs for the exposure variable (Expo) showed similar values for women and for men overall, 1.09 (0.97-1.24) and 1.12 (0.99-1.27) respectively. RRs for two regions were statistically significant higher than one, Canary Islands showed an excess of risk of 1.35 (1.05-1.72) for women and 1.50 (1.18-1.92) for men and Galicia showed an excess of risk of 1.35 (1.04-1.75) for men. Also Madrid showed a RR for women statistically significant lower than one, 0.64 (0.42-0.98), and Valencia showed for men a decrease in risk of 0.69 (0.53-0.91).

**Figure 4:**
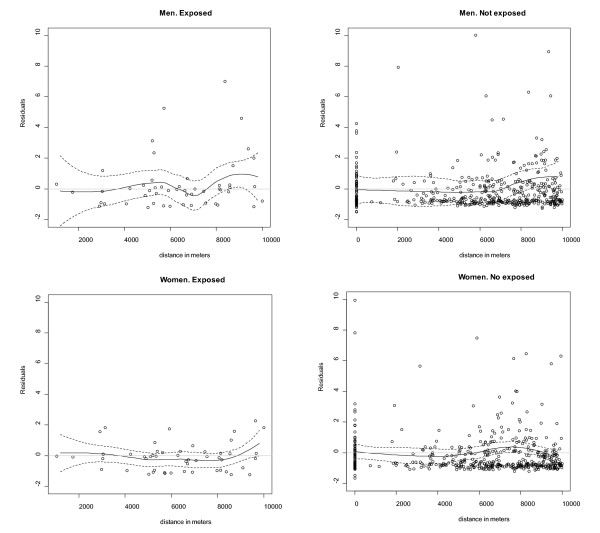
**Graphs of residuals vs distance**. The straight line is the lowess smoother and the dashed lines are point wise 95% confidence bands. For non-exposed areas the represented distance is the distance from the municipal centroid to the centre of the area, dots with distance equal 0 are the municipalities located in the centre of each non-exposed area.

The analyses of the random effects and residuals did not show any discernible trend with distance, nor was there much difference between exposed and non-exposed populations (Figure 4).

## Discussion

The results suggest association between non-Hodgkin lymphoma mortality risk and residence within 10 km of a refinery. Estimated RRs showed around a 10% increased risk for the exposed municipalities for both men and women. For the exploratory analysis with the whole country (8098 municipalities) these overall RRs were statistically significant, whereas after controlling the potential confounders by the matching (528 matched municipalities) they were very close to statistical significance. Two regions showed statistically significant increase in risk with both models, Canary Islands and Galicia; these risks were higher than the risk associated with the exposure variable, reaching around 40%. The analysis of the residuals showed no change in the mortality risk distribution with increasing distance from the municipality of residence to the refinery.

The main strength of this analysis was the control of potential confounding by the use of a matched analysis. Comparing the results between the spatial model and the matched analysis we see that RRs and 95%CIs for men are almost identical for both models, while for women the matched analysis provided lower and not statistically significant RRs. Values for the RRs of the regions were also different; however, Canary Islands and Galicia's men still showed very high and statistically significant risks. These results could suggest that the matched analysis has eliminated part of the confounding that was affecting women but not men.

Another important contribution was the joint study of the 10 refineries in the same analysis. The individual study of each refinery would have required individual data on each case, which was not available for our study. In the study of environmental factors that can be associated with health outcomes the availability of a large data set is an advantage in most situations; nevertheless a naive analysis of such data can produce biased results due to confounding. In this study we initially fitted a spatial model using all Spanish municipalities to perform an exploratory analysis. This analysis provided an initial assessment of the presence of association between NHL mortality and residence in the vicinity of refineries; however, we could not be confident that the large heterogeneity among the municipalities regarding population, socio-demographic characteristics and level of industrialization had been fully controlled by the spatial model.

We therefore performed a matched analysis over a sample of the municipalities. If the matching is accurate, accounting for the matching in the analysis will eliminate confounding by the matching variable [24]. Consequently, we matched geographical areas with the aim of eliminating potential confounders such as socio-demographic status and level of industrialization: Some of the refineries were located in middle size cites and others close to big cities but none in small towns (about 50% of the Spanish municipalities have a population below 5000 inhabitants); and some of the refineries were located in highly industrialized regions implying more sources of industrial pollution. The final number of selected areas provided a reasonable sample size, choosing too many matched unexposed areas could introduce residual confounding into the analysis due to less comparability in term of matching factors. We matched areas, not municipalities, to account for the continuous nature of the pollutant emissions that move through the artificial administrative boundaries.

The main objective of this analysis was to study the links between NHL mortality and exposure to refineries; however, we could not ignore the strong regional variation of the NHL mortality shown in previous studies conducted in Spain [16,25]. The omission of regional data would have generated biased results, and therefore misleading conclusions, given that two of the refineries are located in the regions with higher NHL risk, Canary Islands and Galicia.

We used a mixed effect Poisson model to analyse the matched data because this allows for extra-Poisson variation resulting from unmeasured confounders and misclassification [26]. Previous studies have suggested using conditional Poisson regression models to approach the study of matched data [27]; however, this does not allow extra-Poisson variation.

To the best of our knowledge, this is one of the first point source modelling studies that has used matched geographical areas. We have already mentioned a study conducted in the UK where a highly industrialized region was matched with a similar region without industry according to its socio-economical characteristics [6]; however in our study we have matched several exposed areas with multiple non-exposed and used more data sources in the matching strategy. Nevertheless, limitations of our approach mainly came from the nature and definition of the available data including the ecological nature of the socio-demographic data and the lack of information on specific industrial emissions.

The study used mortality data from the official registers. Unfortunately, at present there is no nationwide cancer register in Spain. The non-inclusion of incidence data is an important limitation on the study of potential risk factors. The lack of information about non-lethal cancer cases may bias the analysis; however, according to Gomez-Perez et al. [28], in Spain relative effects of morbidity associated with tumours that have lower survival rates are well represented by death certificates. Furthermore, we believe there are at most small differences in survival rates or quality of care between regions due to the universal health system established in Spain in 1986. According to the EUROCARE-4 the overall five-year survival rate of NHL for Spain is 51.9% [29].

Another delicate decision was the use of distance as proxy for exposure, which may introduce bias; for an extended discussion about this topic, see for example [9,30]. Yet, we would like to point out that the use of isotropic distance instead of a more general metric may introduce bias in the results; however, these problems would tend to affect the analysis by restricting the ability to find positive results, shifting the results towards the null hypothesis, rather than providing spurious associations. Another important decision in the definition of the exposure variable was the maximum distance of 10 km. The previous studies on refineries and petrochemical plants defined shorter distances but due to the aggregated nature of our data, the spatial distribution of the residential areas in Spain (normally around the town centres with large empty areas between towns), and the large areas occupied by the refinery plants, a smaller buffer would have provided very few exposed individuals.

Another limitation is the use of aggregated data for the exposure variable that implied important assumptions. We assumed that registered place of residence determines the estimated exposure; hence no allowance is made either for long-term movements between different addresses or short-term movements between home and work; instead we considered that the whole municipal population to be exposed to the same type and amount of pollutant substances. These assumptions could introduce a misclassification problem to add to the intrinsic ecological bias present on ecological studies (Ecological fallacy); nevertheless the use of small areas (municipalities) as units reduces the risks of ecological bias and misclassification [31].

The lack of risk gradient with distance is consistent with previous studies in the UK [5]. In this study we consider residence as place of outdoor exposure to refineries emissions. We do not consider occupational exposures, indoor exposures or other outdoor exposures to substances that could be related to a risk increase. All those factors may contribute to non-differential exposure misclassification which can bias the results but also could hinder the detection of a distance effect over the risk of NLH mortality.

The aetiology of NHL is rather poorly understood [32]. The best described risk factor for NHL is immune deficiency. Some theories have associated it with the HIV epidemic [33], though the inclusion of Highly Active Antiretroviral Treatments (HAARTs) does not appear to have affected the rising trend in NHLs [34]. However, these specific infections account for a very small proportion of total NHL incidence. In addition to immune deficiency and infection, other immune-related conditions like rheumatoid arthritis, systemic lupus erythema, Sjogren's syndrome, psoriasis and coeliac disease, are increasingly being recognised as related to NHL risk [35]. A variety of other exposures are less strongly related to NHL risk.

From the chemical exposure point of view, some studies have linked lymphomas to exposure to substances such as agricultural chemicals [34], pesticides [36] and dioxins released by incinerators [37]. Alternatively, a number of occupational exposure studies reported higher NHL incidence and mortality among workers exposed to industrial solvents [38,39]. Two recent meta-analyses of cohort and case-control studies of NHL, benzene and refinery work provided evidence that benzene is associated with NHL [40,41]. Benzene is a known human carcinogen and has been shown to have the ability to produce chromosomal and genetic changes important to NHL induction [1]. Benzene was also linked to lymphomas in several animal studies including the 1986 US National Toxicology Program carcinogenicity bioassay of benzene [42-45].

Previous studies of NHL and environmental exposure to industrial pollution offered opposite results; De Roos et al. [11] did not find association between living near industries and increase in NHL. However, two case-control studies conducted in the US and Canada [8,12] and a study in Spain [9] suggested the existence of association between residence in the proximity of industry and increase in NHL. According to the IARC, the substances associated with NHL are tetrachloroethylene, classified as probably carcinogenic to humans (Group 2A); and ethylene oxide, classify as an agent carcinogenic to humans (Group 1) [1].

Preceding studies that analysed specifically the possible effects of residence near petrochemical plants suggested association, but neither of them was conclusive. De Roos et al. [11] evaluated the risk of NHL associated with residence within 2 miles of industries. Their study included 94 cases and 76 controls living within 2 miles from refineries. Risk for follicular lymphoma showed an association but not statistical significance (OR = 1.1, CI: 0.7-1.9). In a previous case-control study Linos et al. [12] found an increase in NHL risk among those living near petrochemical industry; however this was not statistically significant. That study included 14 cases and 18 controls within 3.2 km from the facilities (OR = 1.5, CI: 0.7-3.2). Our results agree with previous studies showing increased risk; but with estimated RRs were generally closer to statistical significance. In our study the number of cases in the exposed areas was 1,134, 589 men and 545 women, while the number of cases in non-exposed areas was 3933, 2038 men and 1895 women. Though case-control studies and ecological studies are not directly comparable, the more statistically conclusive results presented in this study could be due to the larger number of cases included in both exposed and non-exposed categories.

In our results the most important contribution to mortality risk came from its spatial distribution, as expected from previous studies [16,25]. The excess of risk in the regions of Canary Islands and Galicia and low risk in Madrid were already shown in the atlas of municipal mortality cancer in Spain [16]. This regional variation has also been shown in analyses of cancer incidence. The regional and local cancer registers network (REDECAN) covers the 26.5% of the total population gathering incidence data. A recent study based on these data studied the evolution of the incidence of NHL during the last decades [25]. Four of the 13 registers of the network are located in regions that have refineries within their boundaries. The results of this study showed increased risks for Canary Island, Tarragona (Catalonia) and The Basque Country, while Murcia showed risk below one. Unfortunately, the unknown aetiology of NHL hinders the formulation of theories to explain this regional variation.

The results of our analyses showed similarity in the RRs for the exposure variable for men and women, this fact suggests that environmental risk factors contribute to variation in NHL mortality risk. An examination of the information contained in the E-PRTR for 2007 showed that all the refineries but one reported emissions above the thresholds that determined their inclusion in the registry, for the following heavy metals: chrome, lead, nickel and zinc. Furthermore, 8 facilities reported emissions of arsenic, 7 facilities reported emissions of cadmium, 6 facilities reported emissions of benzene and 4 reported emissions of dioxins and furans (PCDD+PCDF). All the above mentioned compounds but lead, are classified by IARC as agents carcinogenic to humans (Group 1); lead is classified by IARC as probably carcinogenic to humans (Group 2A). In addition two facilities also reported emissions of naphthalene and polychlorinated biphenyls, and a different one reported emissions of vanadium. These three compounds are classified as agents possible carcinogenic to humans (2B) for the IARC [1]. None of the refineries reported emissions of the chlorinated solvent tetrachloroethylene or ethylene oxide. However, refineries can be associated with exposure to many different chemical agents, so this analysis by itself does not provide direct evidence that any single agent is responsible for the observed increase.

## Conclusions

The results suggest a possible increased risk of NHL mortality among populations residing in the vicinity of refineries, but do not show a gradient in relation with increasing distance; however the regional effect is stronger in the Canary Islands and Galicia. In order to confirm or reject these results, it would be of great interest to seek to improve the exposure markers and ascertain precisely what is happening in the environs of each specific installation. In addition, the availability of incidence data would be very useful to study the less aggressive non-lethal NHL cases, which are not included in this study.

Despite all the limitations mentioned above, the innovative design of the present study allows the transference of many of the advantages of case-control studies to ecological studies. Matching geographical areas according to socio-demographic characteristics can be a useful tool in the study of environmental factors in health outcomes.

## Abbreviations

NHL: Non-Hodgkin lymphoma; IARC: International agency for research on cancer; E-PRTR: European pollutant release and transfer register; RR: Relative risk.

## Competing interests

The authors declare that they have no competing interests.

## Authors' contributions

RR developed the idea for this manuscript, geocoded the data, outlined the analyses, conducted statistical analysis and wrote the manuscript. PD outlined the analyses and provided statistical expertise, reviewed and edited the manuscript and contributed to the discussion. EB, JGP, PFN and GLA geocoded the data, reviewed and edited the manuscript and contributed to the discussion. All authors read and approved the final manuscript.

## Supplementary Material

Additional file 1 **Reported pollutant releases from the Spanish in 2007**. Click here for file
